# Clinical epidemiological characteristics and risk factors for severe pertussis in children in Xiamen

**DOI:** 10.1186/s12879-026-13255-0

**Published:** 2026-04-10

**Authors:** Jinqiang Zhang, Dequan Su, Yongjun Xu, Qingqing Lin, Mei Zeng, Zhiqiang Zhuo, He Tian

**Affiliations:** 1 Department of Infectious Diseases, Xiamen Children’s Hospital, Children’s Hospital of Fudan University Xiamen Branch, 92 Yibin Road, Xiamen, 361006 China; 2 Department of Infectious Diseases, Children’s Hospital of Fudan University, 399 Wanyuan Road, Shanghai, 201102 China; 3 Department of Microbiology, Xiamen Children’s Hospital, Children’s Hospital of Fudan University Xiamen Branch, Xiamen, China

**Keywords:** *Bordetella pertussis*, Children, Severe disease, Vaccination, Risk factors, Antibiotic resistance

## Abstract

**Objective:**

Over the past few years, no pertussis outbreaks or significant increases in case numbers have been documented in Xiamen, and related academic research remains scarce. To address this knowledge gap, this study conducted a retrospective analysis of the epidemiological characteristics of pediatric pertussis in Xiamen in 2024, as well as the clinical features and risk factors for severe cases, This work aims to provide empirical evidence for optimizing local pertussis prevention and control strategies and clinical case management.

**Methods:**

Epidemiological characteristics were analyzed by collecting demographic information on all confirmed pertussis cases admitted to Xiamen Children’s Hospital from January to December 2024 through the hospital’s infectious disease reporting system. Clinical data for hospitalized patients were extracted from electronic medical records to identify risk factors associated with severe pertussis. During outbreak periods, nasopharyngeal swab specimens were collected from selected hospitalized patients and their caregivers for real-time polymerase chain reaction (PCR) detection. A subset of these specimens was subjected to *Bordetella pertussis* culture, and the isolated strains were further subjected to antimicrobial susceptibility.

**Results:**

A total of 287 confirmed pertussis cases were reported in 2024. The median age of the patients was 5.00 years (interquartile range [IQR]: 0.33–7.00 years), with children aged 6 years and older accounting for 45.30%(130/287). Among all cases, 126 (43.90%) were hospitalized, with a median age of 0.46 years (IQR: 0.17–5.00 years), and 17 (13.49%) of these hospitalized cases were classified as severe, with a median age of 0.08 years (IQR: 0.08–0.21 years). Among the 126 hospitalized cases, 51 (40.48%) had possible household exposure; 89(71.43%) presented with paroxysmal coughing; 31(25.40%) experienced post-tussive emesis and 16 (12.70%) had inspiratory whoop; 68 (53.97%) manifested cyanosis or facial flushing during coughing and 4 (3.17%) had apnea; 28 (22.22%) had fever. A total of 58 cases (46.03%) had no documented pertussis vaccination history. Multivariate logistic regression analysis identified unvaccinated status and elevated white blood cell count (> 20 × 10⁹/L), as independent risk factors for severe pertussis. In vitro drug susceptibility testing of 25 *Bordetella pertussis* isolates demonstrated high-level resistance to azithromycin but sensitivity to trimethoprim-sulfamethoxazole, levofloxacin, doxycycline, ceftazidime and cefoperazone/sulbactam.

**Conclusion:**

During the 2024 pertussis epidemic in Xiamen, school-age children aged 6 years and older constituted the most affected population. However, severe pertussis occurred predominantly in unvaccinated infants under 6 months of age. Therefore, timely completion of the primary pertussis vaccination series in infants remains a key strategy for preventing severe pertussis. Additionally, supplementary vaccination for school-age children is necessary to boost waning immunity. Macrolide antibiotics is not advised for empirical treatment of pertussis in China.

**Clinical trial number:**

Not applicable.

## Introduction

Pertussis is an acute respiratory infectious disease caused by *Bordetella pertussis*. It is highly contagious, with general susceptibility in the population [1]. Since the global implementation of pertussis vaccination in 1974, the incidence and mortality rates of pertussis have decreased significantly worldwide [2]. However, since the 1990s, a “pertussis resurgence” has been reported in countries and regions with high vaccination coverage, and the affected population has gradually shifted to older children and adults [1]. In China, the diphtheria-tetanus-whole-cell pertussis vaccine (DTwP) has been included in the national immunization program since 1978. In 2012, DTwP was replaced by the diphtheria-tetanus-acellular pertussis vaccine (DTaP), and a 4-dose immunization schedule was subsequently implemented for children at 3, 4, 5, and 18 months of age. Since 2013, China has witnessed an upward trend in pertussis incidence, which increased from 0.12 per million population in 2013 to 28.9 per million population in 2023. A nationwide pertussis outbreak occurred in China in 2024, with a total of 494,321 reported cases and 31 reported deaths [3]. Pertussis has re-emerged as a major public health concern in China.

Although resistance of *Bordetella pertussis* to macrolide antibiotics has been reported globally, high prevalence of macrolide resistance in *Bordetella pertussis* strains has been documented in multiple regions of China since 2013 [4–7]. Xiamen, located in the economically developed southern region of China, has limited research on pediatric pertussis. During the 2024 nationwide pertussis outbreak, Xiamen also experienced a significant increase in reported cases. Therefore, we retrospectively analysed the clinical characteristics of pediatric pertussis cases reported from a tertiary children’s hospital in Xiamen in 2024, explored the risk factors for severe pertussis, and determined the in vitro resistance patterns of circulating strains. This study aims to provide scientific evidence for formulating local pertussis prevention and control strategies and optimizing clinical management.

## Materials and methods

### Data collection

The pediatric pertussis cases were reported from the Xiamen Children’s Hospital between January and December 2024. The Demographic information and the date of disease onset were collected through the hospital’s infectious disease information reporting system. Relevant clinical data were retrieved from the hospital electronic medical record system, with the following categories included: (1) demographic and clinical baseline data: vaccination history, underlying medical conditions and clinical symptoms; (2) laboratory and imaging findings: complete blood count, inflammatory markers (C-reactive protein, procalcitonin), nasopharyngeal swab PCR and Bordetella pertussis bacterial culture, PCR and culture assays of nasopharyngeal aspirates or sputum specimens targeting respiratory viruses, *Mycoplasma pneumoniae* and Chlamydia, plus imaging results; (3) clinical management records: antimicrobial treatment regimens, oxygen therapy, and intensive care unit (ICU) admission status. We cross-checked the vaccination status reported by parents or documented in electronic medical records against data from the local immunization information system. Household exposure history was recorded as a binary variable (presence/absence of a family member with cough prior to the patient’s onset). Detailed information regarding the duration of exposure, clinical course of the index case, and laboratory confirmation of the source contact was not systematically collected for all patients. Vaccination history was retrieved from the hospital’s electronic medical records and the regional immunization registry, with no missing data among hospitalized cases. Routine laboratory tests, including complete blood count, were performed for all admitted patients; therefore, white blood cell (WBC) and lymphocyte ratio data were available for all 126 hospitalized cases. Other variables (e.g., inflammatory markers) with occasional missing values were excluded from the primary risk factor analysis.This study was approved by the Ethics Committee of Xiamen Children’s Hospital (Approval No.: Xiamen Children’s Hospital Ethics Review [2025] No. 54), with informed consent from patients and parents waived.

### Case definition

All reported pertussis cases were confirmed by the presence of cough symptoms and positive Bordetella pertussis nucleic acid detection via polymerase chain reaction (PCR). For this clinical epidemiological analysis, all included patients had a cough duration of at least 14 days. Clinically diagnosed cases without laboratory confirmation were excluded. The diagnostic criteria for severe pertussis required the presence of any of the following: (1) Recurrent apnea requiring monitoring and oxygen therapy; (2) Severe pneumonia complicated with hypoxemia (SpO₂ < 92% in room air) requiring oxygen supplementation or respiratory support; (3) Pertussis encephalopathy; (4) Pulmonary hypertension; (5) Cardiopulmonary insufficiency; (6) Admission to the intensive care unit (ICU) due to pertussis, in accordance with institutional pediatric intensive care unit (PICU) criteria (e.g., requirement for mechanical ventilation, high-flow oxygen therapy > 2 L/kg/min, recurrent apnea, or hemodynamic instability) [8].

### Laboratory testing methods for *Bordetella pertussis* detection

#### Specimen collection

The attending physician collected a nasopharyngeal swab from the patient for *Bordetella pertussis* testing. Some hospitalized cases also underwent *Bordetella pertussis* bacterial culture. For hospitalized cases, nasopharyngeal washings or sputum samples were also collected within 24 h of admission for nucleic acid detection of multiple respiratory viruses, *Mycoplasma pneumoniae*, and *Chlamydia* (using respiratory pathogen detection kits produced by Ningbo Haier Gene Technology Co., Ltd.), as well as respiratory bacterial culture.

#### PCR detection for *Bordetella pertussis*

Nasopharyngeal swab specimens were tested using a commercial Bordetella pertussis nucleic acid detection kit (PCR-fluorescent probe assay; Shenzhen Yilifang Biotechnology Co., Ltd.) in accordance with the manufacturer’s instructions. The IS481 insertion sequence was used as the amplification target, a standard genetic marker for Bordetella pertussis identification. A cycle threshold (Ct) value ≤ 38 was defined as a positive result.

#### Bacterial culture and quality control for *Bordetella pertussis*

Nasopharyngeal swabs were collected and immediately inoculated onto charcoal agar (OXOID, UK) supplemented with 10% defibrinated sheep blood and *Bordetella* selective supplement (OXOID, UK) for selective isolation of *Bordetella pertussis*. Minimum inhibitory concentrations (MICs) of antimicrobial agents against clinical isolates were determined using gradient test strips (Liofilchem, Italy) [6]. As standardized interpretive criteria for *Bordetella pertussis* antimicrobial susceptibility have not been defined by the Clinical and Laboratory Standards Institute (CLSI) or European Committee on Antimicrobial Susceptibility Testing (EUCAST), the following breakpoints were adopted in this study: azithromycin, trimethoprim‑sulfamethoxazole, levofloxacin, and doxycycline were interpreted using breakpoints for *Haemophilus influenzae*; ceftazidime and cefoperazone/sulbactam used breakpoints for *Escherichia coli*. All interpretations followed the 2024 criteria from CLSI [9] and the Chinese Committee on Antimicrobial Susceptibility Testing (CCAST, 2024) [10].

Strict quality control (QC) was performed throughout the experiments. *Bordetella pertussis* ATCC 9797 was included as the QC strain in each batch of culture and susceptibility testing to validate assay performance. Because no official QC ranges are currently available for *Bordetella pertussis*, acceptable QC MIC ranges were referenced from CLSI M100 and CCAST guidelines for *Haemophilus influenzae* (azithromycin, trimethoprim‑sulfamethoxazole, levofloxacin, doxycycline) and *Escherichia coli* (ceftazidime, cefoperazone/sulbactam). All QC results were within acceptable ranges, confirming the reliability of the experimental data.

### Statistical methods

Data analysis was performed using SPSS 30.0 statistical software. For normally distributed quantitative data, results are expressed as mean ± standard deviation (Mean ± SD). Intergroup comparisons were performed using t-tests, or adjusted t-tests when variances were unequal. For non-normally distributed data, results are expressed as median (25th and 75th percentiles): median (IQR). Intergroup comparisons were performed using the Mann-Whitney U rank-sum test. Count data were expressed as case numbers (n) and percentages (%). Intergroup comparisons were performed using chi-square tests or Fisher’s exact tests. Multivariate analysis was conducted via logistic regression. The significance level was set at α = 0.05, with *P* < 0.05 indicating statistically significant differences.

## Results

### Characteristics of pertussis outbreak

**Fig. 1:**
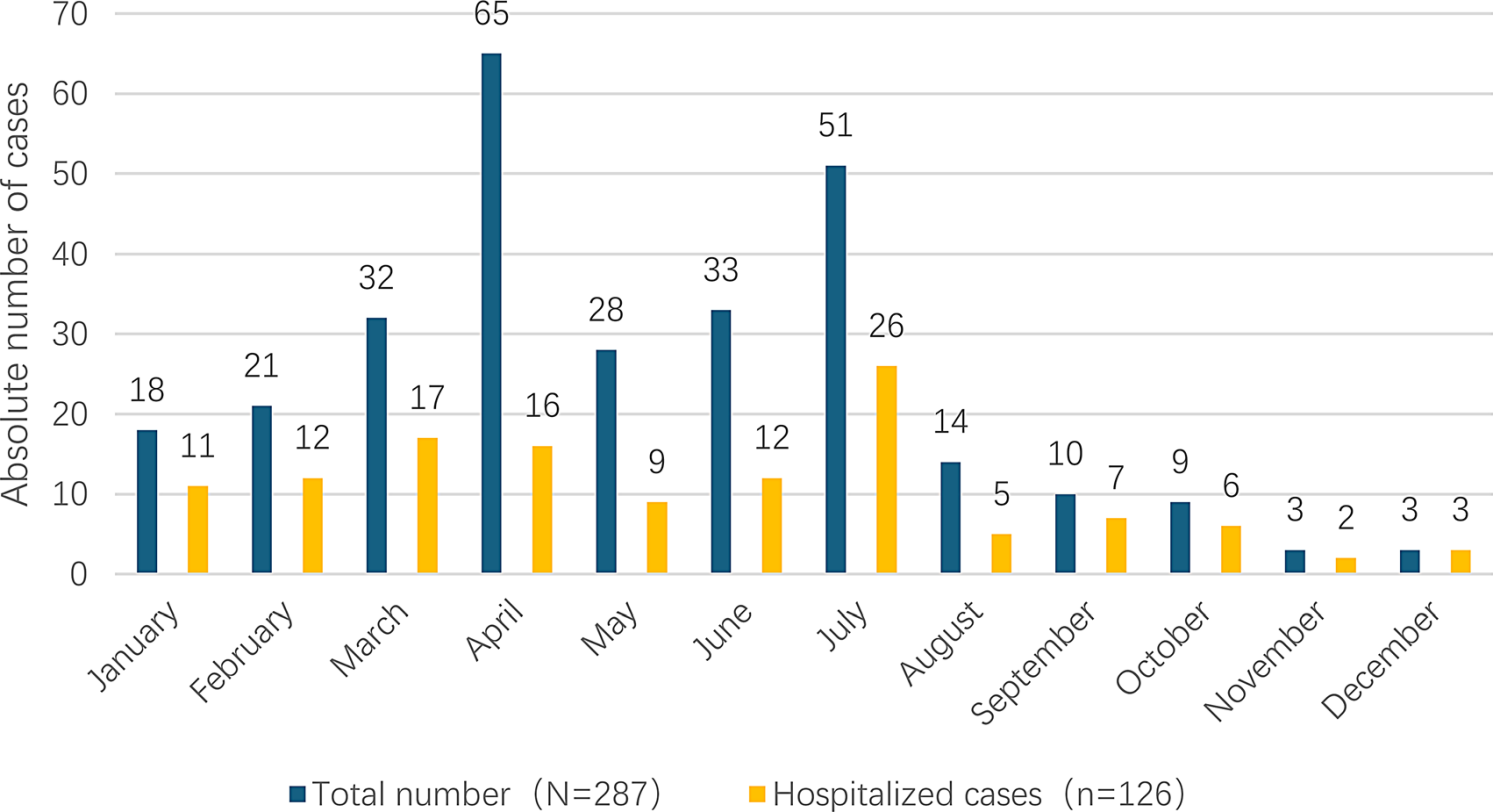
Total number of confirmed and hospitalized *Pertussis* cases in Xiamen, January–December 2024

**Fig. 2:**
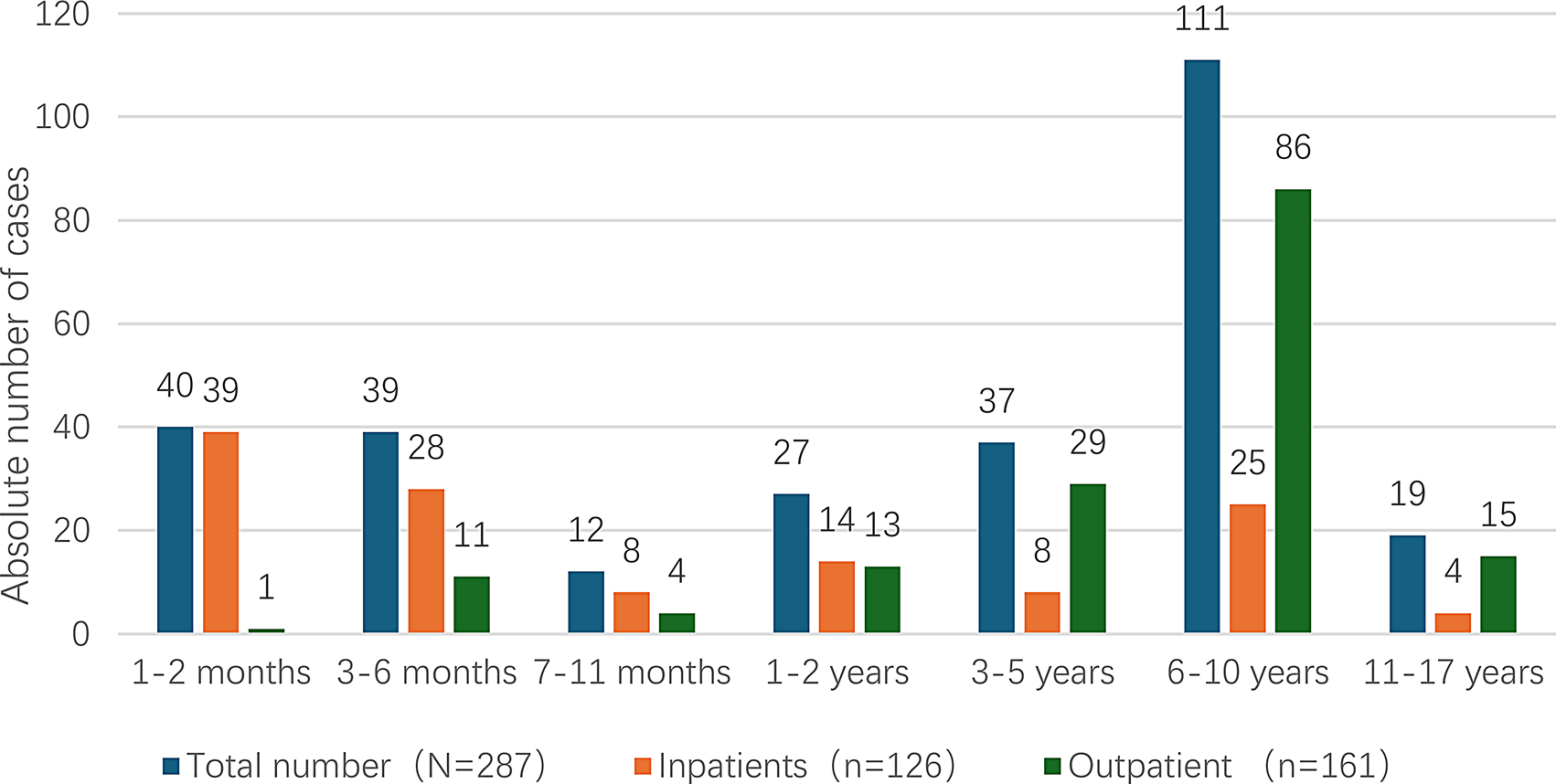
Age distribution of 287 confirmed *Pertussis* cases in Xiamen (Including 126 Inpatients and 161 Outpatients)

From January to December 2024, pertussis cases occurred throughout the year, with the patients concentrated between March and July, accounting for 72.82% of the total cases (see Fig. 1). A total of 287 PCR-confirmed pediatric pertussis cases were reported, including 162 (56.45%) were male and 125 (43.55%) were female; the median age was 5.00 years (IQR: 0.33–7.00 years, range: 12 days–15 years). The age distribution was as follows: 40 cases (13.94%) were < 3 months, 39 cases (13.59%) were 3–6 months, 12 cases (4.18%) were 6–11 months, 27 cases (9.41%) were 1–2 years, 37 cases (12.89%) were 3–5 years, 111 cases (38.68%) were 6–10 years, and 19 cases (6.62%) were 11–15 years. Children aged 6 years and older accounted for 130 cases (45.30%) (see Fig. 2).

### Clinical characteristics of hospitalized children with pertussis

A total of 126 children (43.90%) were hospitalized, including 74 males (58.73%) and 52 females (41.27%), with a male-to-female ratio of 1.42:1. The median age of the hospitalized children was 0.46 years (IQR: 0.17, 5.00), ranging from 12 days to 13 years. Age distribution was as follows: 39 cases (30.95%) were < 3 months, 28 cases (22.22%) were 3–6 months, 8 cases (6.35%) were 6–11 months, 14 cases (11.11%) were 1–2 years, 8 cases (6.35%) were 3–5 years, 25 cases (19.84%) were 6–10 years, and 4 cases (3.17%) were 11–15 years (see Fig. 2).

A total of 58 cases (46.03%) had no documented pertussis vaccination history; which was divided into two subgroups: individuals who met the official vaccination criteria but remained unvaccinated, and those who were not yet of the eligible age for pertussis immunization. Among the latter subgroup, children aged < 3 months, 3–6 months, and > 6 months accounted for 68.97%, 29.31%, and 5.17%, respectively. Fifty-one cases (40.48%) had a clear history of exposure to household members with cough prior to onset. Notably, nasopharyngeal swabs were simultaneously collected from 55 family members accompanying the 31 hospitalized patients for Bordetella pertussis real-time PCR. Strikingly, 34 of these household contacts tested positive, corresponding to a high positivity rate of 61.82%.

Eighty-nine (70.63%) cases presented with paroxysmal coughing, 31 (24.60%) cases had post-tussive vomiting, 16 (12.70%) cases had inspiratory whoop, 68 (53.97%) cases developed cyanosis or facial flushing during coughing, and 4 (3.17%) cases experienced apnea. Fever was observed in 28 cases (22.22%). Laboratory examinations showed that 31 cases (24.60%) had elevated peripheral blood white blood cell counts (20 × 10⁹/L ~ 46.59 × 10⁹/L), and 66 (52.38%) cases showed increased peripheral blood lymphocyte ratios (> 60%).

Seventy-eight cases (61.90%) had co-detection of other pathogens, including 74 cases (58.73%) with respiratory virus co-detection, 24 cases (19.05%) with bacterial co-detection, and 11 cases (8.73%) with *Mycoplasma pneumoniae* co-detection. Twenty cases (15.87%) had co-detection of two or more pathogens.

All 126 hospitalized pertussis patients underwent chest imaging; pneumonia was confirmed in 84 (66.66%) cases, and 13 cases (10.32%) showed pulmonary consolidation and/or atelectasis. Sixteen cases (12.70%) received oxygen therapy, including 3 cases (2.38%) who underwent high-flow oxygen therapy and 1 (0.79%) case requiring mechanical ventilation. Nineteen cases (15.08%) were administered methylprednisolone and 16 (12.70%) cases received gamma globulin therapy. All 126 hospitalized patients achieved clinical improvement and were discharged.

### Comparison of clinical characteristics between severe and non-severe pertussis cases in children

Among 126 hospitalized pertussis patients, 17 cases (13.49%, 17/126) were severe. The patients were divided into non-severe (*n* = 109) and severe (*n* = 17) groups. Compared with the non-severe group, the severe group had a younger age at onset, higher proportions of paroxysmal cough, post-tussive vomiting, cyanosis or facial flushing, a higher rate of unvaccinated status, more significant elevations in white blood cell count and lymphocyte ratio, and a higher rate of bacterial co-infection. All these differences between the two groups were statistically significant (*P* < 0.05) (see Table 1).

**Table 1:** Comparison of clinical characteristics between non-severe and severe groups in *Pertussis *(total number: *N* = 126)

Characteristics	Non- Severe (*n* = 109)	Severe (*n* = 17)	χ^2^/ Z	*P* value
Gender(Male/Female)	63/46	11/6	0.29	0.59
Age [y, M (*P*_25_, *P*_75_)]	0.83 (0.25, 6.00)	0.08(0.08, 0.21)	44.01	<0.05
Length of hospital stay(days)	7.64 ± 3.16	16.47 ± 6.17	19.83	<0.05
Duration of cough before admission (days)	11.38 ± 8.60	11.53 ± 13.22	0.13	0.95
Exposure history	41	10	2.75	0.10
**Clinical Features**				
Paroxysmal spasmodic cough	73(66.97%)	16(94.12%)	5.22	<0.05
Whoopjing sound	13(11.93%)	3(17.65%)	0.43	0.51
Post-tussive vomiting	22(20.18%)	10(58.82%)	11.59	<0.05
Apnea	3(2.75%)	1(5.88%)	0.47	0.49
Cyanosis or facial flushing during coughing	52(47.71%)	16(94.12%)	12.75	<0.05
Fever	23(21.10%)	5(29.41%)	0.59	0.44
Unvaccinated status	42(38.53%)	16(94.12%)	18.29	<0.05
**Laboratory tests**				
WBC>20 × 10^9^/L	22 (20.18%)	11 (64.71%)	15.08	<0.05
Lymphocyte ratio(>60%)	53(48.62%)	13(70.59%)	5.27	<0.05
CRP(>8 mg/l)	18(16.51%)	0(0.00%)	3.28	0.07
PCT(>0.5ng/l)	4(23.53%)	0(0.00%)	0.64	0.42
Pneumonia(PCT > 0.5 ng/mL)	71(65.14%)	13(76.43%)	0.85	0.36
Pulmonary consolidation (atelectasis)	13(11.93%)	3(17.65%)	0.43	0.51
Co-infection with viruses	63(57.80%)	11(64.71%)	0.29	0.59
Co-infection with bacteria	17(15.60%)	7(41.18%)	6.24	<0.05

P25, P75: 25th and 75th percentiles; total number ༚including both severe and non-severe cases

### Multivariate risk factor analysis for severe pertussis in children

Multivariate Risk Factor Analysis revealed that unvaccinated status (OR = 8.389, 95% CI: 1.249–18.511, *P* = 0.027) and WBC > 20 × 10⁹/L (OR = 6.373, 95% CI: 1.500–16.764, *P* = 0.026), are independent risk factors for severe pertussis (see Table 2).

**Table 2:** Analysis of risk factors for severe pertussis in children (total number: *N* = 126)

Clinical characteristics / Laboratory findings	B	S.E.	Wald	*P*	OR	95% CI
Unvaccinated status	3.566	1.613	4.891	0.027	35.389	1.500–16.764
WBC (>20 × 10^9^/L)	1.852	0.831	4.962	0.026	6.373	1.249–18.511

WBC: white blood cell; DtaP: diphtheria-tetanus-acellular pertussis vaccine; B: Regression Coefficient; S.E.: Standard Error; OR: odds ratio; CI: confidence interval

### In vitro antimicrobial resistance

A total of 25 *Bordetella pertussis* were isolated, with a positive culture rate of 19.84% (25/126). All the 25 (100%) were resistant to azithromycin, with minimum inhibitory concentrations (MIC) > 256.000 µg/mL; all isolates were susceptible to ceftazidime and cefoperazone/sulbactam, with MICs < 0.064 µg/mL; and all isolates were susceptible to levofloxacin, doxycycline, and trimethoprim-sulfamethoxazole (TMP-SMZ), with MICs of 0.25 µg/mL, 0.5 µg/mL, and 0.016/0.304 µg/mL, respectively. (See Table 3).

**Table 3:** Antimicrobial resistance profiles of 25 *Bordetella pertussis* isolates from Xiamen Children’s Hospital in 2024

Antimicrobial agent	Minimal Inhibitory Concentrations (MICs, µg/ml)			Interpretive category [Number (%)]		
	MIC50	MIC90	Range	Susceptible	Intermediate	Resistant
Azithromycin	> 256	> 256	> 256	0 (0)	0 (0)	25 (100)
Trimethoprim-sulfamethoxazole	0.016/0.304	0.125/2.375	≤ 0.008/0.152-2/38	25 (100)	0 (0)	0 (0)
Ceftazidime	≤ 0.064	≤ 0.064	≤ 0.064	25 (100)	0 (0)	0 (0)
Cefoperazone /Sulbactam	≤ 0.064	≤ 0.064	≤ 0.064	25 (100)	0 (0)	0 (0)
Levofloxacin	0.25	0.25	0.125-0.5	25 (100)	0 (0)	0 (0)
Doxycycline	0.5	1	0.25-1	25 (100)	0 (0)	0 (0)

## Discussion

This study reveals that the largest number of pertussis cases occurred among school-age children aged 6–10 years, accounting for 38.68% of the total reported cases. However, young infants under 6 months of age still accounted for 23.34% of the cases. Furthermore, infants under 6 months of age constituted 53.17% of hospitalized cases. National surveillance data indicate that the annual reported number of cases in 2024 was approximately 12 times higher than that in 2023 [3]. Although official historical surveillance data on pertussis is not available in Xiamen, we observed a marked increase in the number of pertussis cases in 2024 compared with previous years, with a peak occurring during the spring and summer months (April to July). This trend is consistent with the national incidence peak during the same period [3]. Surveillance studies in developed countries have shown that an epidemic cycle of 2–5 years exists in developed countries with high acellular pertussis vaccine coverage [11]. Currently, data from China’s passive surveillance system cannot fully reflect the cyclical patterns of pertussis epidemics in recent years and thus requires improvement.The highest pertussis incidence among children aged 6–10 years may be associated with the waning of vaccine-induced immunity, considering that this age group is 4–8 years after primary vaccination, which is beyond the peak period of vaccine efficacy reported in previous studies. However, in the absence of serological data on antibody titers or PCR-confirmed reinfection records, this association remains speculative.

Since 2009, the coverage rate of China’s three-dose primary immunization with diphtheria-tetanus-pertussis (DTP) vaccine has remained consistently above 99% [12]. This study shows that school-age children aged 6–10 years accounted for the majority of pertussis cases, all of whom had completed the three-dose primary DTP vaccination series and one booster dose. However, vaccine-induced antibody levels gradually decline with age, compromising protective efficacy. Domestic studies have shown that the protective effect induced by the pertussis vaccine is short-term, reaching its lowest level by 6 years of age, with immunity waning 3–5 years after vaccination [13]. In China, seroepidemiological studies have demonstrated that children aged 1–2 years have the highest positivity rate of pertussis toxin (PT)-IgG (81.4%), which then decreases to 67.7% in those aged 3–4 years and drops sharply to 9.8% in those aged 5–6 years. The PT-IgG positivity rate reaches its lowest point (4.7%) in children aged 10–14 years [14]. Our field findings and domestic seroepidemiological data suggest that it is necessary to administer at least one additional booster dose of pertussis vaccine to school-age children aged 5–6 years. Following the large-scale pertussis outbreak, China’s pertussis vaccination schedule has been optimized, with the fifth dose added to the schedule since January 2025. In addition, we found that 40.48% of hospitalized pediatric pertussis cases had suspected exposure to family members with cough symptoms. Furthermore, we confirmed that *Bordetella pertussis* was detected in 61.82% of accompanying family caregivers. The high positivity rate of 61.82% among household contacts underlines the importance of close contact screening for *Bordetella pertussis* infections in adults are largely neglected and underdiagnosed. In developed countries experiencing pertussis resurgence, an increase in pertussis cases among adolescents and adults has been observed [15, 16]. However, reported cases of pertussis in adolescents and adults remain scarce in China. Pertussis cases in adolescents and adults are often characterized by milder and atypical symptoms, leading to underdiagnosis [17, 18]. Enhanced active symptom-based surveillance in these susceptible populations is warranted to fully understand the epidemiological profile of pertussis in China.

Data analysis revealed significant differences in clinical characteristics between the severe and non-severe pertussis groups. The proportion of children under 3 months of age was significantly higher in the severe group than in the non-severe group. Infants younger than 3 months face a high risk of disease progression to severe cases, which is consistent with findings from existing studies [19–24]. Among the 17 severe cases, 15 were unvaccinated infants under 3 months of age, indicating that lack of vaccination is a primary risk factor for severe pertussis. Vaccination remains the most effective measure for preventing pertussis, particularly severe disease. A study from Switzerland demonstrated that the effectiveness of the pertussis vaccine against hospitalization increases significantly after each completed dose of the acellular pertussis vaccine, with effectiveness rates of 42.1%, 83.9%, 98.2%, and 100% after the first, second, third, and fourth doses, respectively [25]. Therefore, in the era of pertussis resurgence, completing the 3-dose primary pertussis vaccination series remains the most critical measure for preventing severe pertussis in infants. Starting in January 2025, China has revised the DTaP vaccine schedule in two key aspects: changing the primary series administration time from 3, 4, and 5 months of age to 2, 4, 6 months of age in infants. This schedule revision will undoubtedly provide earlier protection for young infants and is expected to reduce severe pertussis cases among infants under 3 months of age. Thus, priority strategies for preventing severe pertussis in infants and young children include improving adherence to timely pertussis vaccination and promoting maternal vaccination during pregnancy to achieve maternal-fetal protection in China in the future.

This study further demonstrated that the proportion of hospitalized children with severe pertussis having peripheral blood white blood cell counts exceeding 20 × 10⁹/L, was significantly higher than that in the non-severe group, and this phenomenon was most commonly observed in infants under 6 months of age. This finding is consistent with previous studies [26–28], indicating that a marked elevation in peripheral blood WBC counts is closely associated with disease severity. The underlying mechanism involves pertussis toxin (PT) interfering with lymphocyte migration and adhesion processes, which obstructs the migration of lymphocytes from the blood to tissues and consequently causes an abnormal increase in the proportion of peripheral blood lymphocytes [29]. Leukocytosis can serve as a warning indicator for severe pertussis, as aggressive hyperleukocytosis may lead to pulmonary hypertension and cardiopulmonary failure.

Since 2013, a high prevalence of macrolide resistance in *Bordetella pertussis* has been reported in China. Specifically, the macrolide resistance rate among *Bordetella pertussis* strains in Beijing and Shanghai approached nearly 100% during 2022–2023 [28, 30, 31]. Antibiotic susceptibility testing conducted in this study demonstrated that all *Bordetella pertussis* isolates from Xiamen were resistant to azithromycin. Owing to the paucity of prior data on pertussis antibiotic resistance in Xiamen, the majority of hospitalized patients enrolled in this study received empirical azithromycin treatment. Early administration of effective antibiotic therapy is crucial for improving clinical outcomes and reducing the incidence of severe complications. This study provides novel evidence regarding azithromycin resistance in *Bordetella pertussis* isolates from Xiamen. The 2024 Chinese Guidelines for the Diagnosis and Treatment of Pertussis recommend trimethoprim-sulfamethoxazole (TMP-SMZ) as the first-line treatment for pertussis in children aged 2 months and older. For infants younger than 2 months, intravenous cefoperazone/sulbactam may be considered [3, 32]. Considering the in vitro susceptibility of *Bordetella pertussis* to levofloxacin and doxycycline, these agents may serve as alternative therapeutic options for patients with contraindications to or for whom caution is advised when using TMP-SMZ. A recent prospective observational study reported that levofloxacin is non-inferior to TMP-SMZ in the treatment of pertussis [33]. All *Bordetella pertussis* isolates in this study displayed high-level resistance to azithromycin. While the molecular mechanisms (e.g., 23 S rRNA mutations) were not characterized herein, previous studies conducted in China have confirmed that such phenotypic resistance is predominantly associated with the A2047G mutation in the 23 S rRNA gene [6]. This consistent national resistance pattern strongly supports the avoidance of macrolides for the empirical treatment of pertussis.

This study has several limitations. First, it was a single-center retrospective study, with all cases recruited from a single children’s hospital. This design may introduce selection bias and limit the generalizability of the findings. Second, the absence of a control group of mild community cases or healthy children precludes accurate estimation of pertussis vaccine effectiveness in preventing infection or reducing hospitalization risk, and also hinders precise quantification of the association between risk factors such as household exposure and infection outcomes. Furthermore, due to the small number of severe cases (*n* = 17), the logistic regression model was considered exploratory. This may result in model overfitting and excessively wide confidence intervals. To avoid overfitting, a reduced model containing only clinically essential variables was employed.

## Conclusion

This study addresses the local knowledge gap regarding the epidemiological profiles of pertussis, providing evidence for pertussis prevention, control, and case management in Xiamen. Future efforts should enhance active screening and testing for pertussis in outpatient settings of both pediatric and adult hospitals among patients presenting with paroxysmal coughing or cough lasting for more than two weeks. Concurrently, following the revision of China’s pertussis vaccination schedule, priority should be given to monitoring severe pertussis cases in infants and pertussis cases in school-aged children and adolescents.

## Data Availability

The datasets used and/or analysed during the current study are available from the corresponding author on reasonable request.

## Abbreviations

IQR: Interquartile range
WBC: White blood cell
CRP: C-reactive protein
PCT: Procalcitonin

## References

[1] Liu Y, Yu D, Wang K, Ye Q. Global resurgence of pertussis: A perspective from China. J Infect. 2024;89(5):106289. 39357571 10.1016/j.jinf.2024.106289

[2] Zhu Y, Zhang W, Hu J, Luo S, Zhou Y, Tang X, Yan R, Deng X, He H. Seroprevalence of IgG antibodies against pertussis toxin in the Chinese population: A systematic review and meta-analysis. Hum Vaccin Immunother. 2024;20(1):2341454. 38695296 10.1080/21645515.2024.2341454 PMC11067989

[3] National Disease Control and Prevention Administration. China CDC. National notifiable infectious diseases. http://www.ndcpa.gov.cn/. Accessed 5 Sept 2025.

[4] Zhou G, Li Y, Wang H, Wang Y, Gao Y, Xu J, Wang F, Peng T, Zhang M, Shao Z. Emergence of Erythromycin-Resistant and Pertactin- and Filamentous Hemagglutinin-Deficient *Bordetella pertussis* Strains - Beijing, China, 2022–2023. China CDC Wkly. 2024;6(20):437–41. 38846358 10.46234/ccdcw2024.085 PMC11150165

[5] Wu X, Du Q, Li D, Yuan L, Meng Q, Fu Z, Xu H, Yao K, Zhao R. A Cross-Sectional Study Revealing the Emergence of Erythromycin-Resistant *Bordetella pertussis* Carrying ptxP3 Alleles in China. Front Microbiol. 2022;13:901617. 35923401 10.3389/fmicb.2022.901617 PMC9342848

[6] Cai J, Chen M, Liu Q, Luo J, Yuan L, Chen Y, Chen M, Zeng M. Domination of an emerging erythromycin-resistant ptxP3 *Bordetella pertussis* clone in Shanghai, China. Int J Antimicrob Agents. 2023;62(1):106835. 37127126 10.1016/j.ijantimicag.2023.106835

[7] Feng Y, Chiu CH, Heininger U, Hozbor DF, Tan TQ, von Konig CW. Emerging macrolide resistance in *Bordetella pertussis* in mainland China: Findings and warning from the global pertussis initiative. Lancet Reg Health West Pac. 2021;8:100098. 34327426 10.1016/j.lanwpc.2021.100098 PMC8315362

[8] Yao K, Li L. Advances in the diagnosis of severe pertussis and in the research of mortality risk factors from pertussis. Chin J Appl Clin Pediatr. 2019;34(22):1681–5.

[9] Clinical and Laboratory Standards Institute (CLSI). Performance standards for antimicrobial susceptibility testing. 34th ed. CLIS supplement M100 (ISBN 978-1-68440-220-5 [Print]; (ISBN 978-1-68440-221-2 [Electronic]). Clinical and Laboratory Standards Institute, USA, 2024.

[10] Hu FP, Guo Y, Wang MG. Performance standards and typical report interpretation for bacterial susceptibility testing. 2nd ed. Shanghai Scientific and Technical Publishers. [ISBN 978-7-5478-6602-3]; 2024.

[11] Who. Pertussis vaccines: WHO position paper. August 2015–Recommendations Vaccine. 2016;34(12):1423–5. 26562318 10.1016/j.vaccine.2015.10.136

[12] WHO. Diphtheria tetanus toxoid and pertussis (DTP) vaccination coverage [OB/OL]. https://immunizationdata.who.int/pages/coverage/dtp.html?CODE=CHN&ANTIGEN=&YEAR=. Accessed 20 May 2025.

[13] He H, Zhu Y, Jin M, Zhou Y, Tang X, Yan R, Deng X, Chen K. The decline in immunity and circulation of pertussis among Chinese population during the COVID-19 pandemic: A cross-sectional sero-epidemiological study. Vaccine. 2022;40(48):6956–62. 36283895 10.1016/j.vaccine.2022.10.020 PMC9581792

[14] Wang S, Zhang S, Liu J. Resurgence of pertussis: Epidemiological trends, contributing factors, challenges, and recommendations for vaccination and surveillance. Hum Vaccin Immunother. 2025;21(1):2513729. 40491090 10.1080/21645515.2025.2513729 PMC12153400

[15] UK Health Security Agency. Confirmed cases of pertussis in England by month, to end October 2024. https://www.gov.uk/government/publications/pertussis-epidemiology-in-england-2024/confirmed-cases-of-pertussis-in-england-by-month.

[16] Australian Government Department of Health and Aged Care. National notifiable disease surveillance system. Commonwealth of Australia, Department of Health. Accessed 10 May 2025. https://nindss.health.gov.au/pbi-dashboard/.

[17] Zhang Z, Wang Q, Zhu Q, Bai S, Liu Y, Ren J, Xu X, Qu J, Pan J, Lu L, et al. Seroepidemiology of pertussis immunity in five provinces of China: A population-based, cross-sectional study. Hum Vaccin Immunother. 2024;20(1):2417532. 39544177 10.1080/21645515.2024.2417532 PMC11572084

[18] MacIntyre CR, de Sousa JC, Heininger U, Kardos P, Konstantopoulos A, Middleton D, Nolan T, Papi A, Rendon A, Rizzo A, et al. Public health management of pertussis in adults: Practical challenges and future strategies. Hum Vaccin Immunother. 2024;20(1):2377904. 39016172 10.1080/21645515.2024.2377904 PMC11259069

[19] Wang C, Zhang H, Zhang Y, Xu L, Miao M, Yang H, Liu Y, He S, Pang L. Analysis of clinical characteristics of severe pertussis in infants and children: a retrospective study. BMC Pediatr. 2021;21(1):65. 33546645 10.1186/s12887-021-02507-4 PMC7863367

[20] Liu J, Lu X, Zhu D, Huang J, Zang P, Xiao Z, Zhang X, Chen Y, Luo H, Zeng X. Clinical features of pertussis and risk factors of severe pertussis in children. Chin Pediatr Emerg Med. 2022;10(29):796–802.

[21] Hu Y, Liu Q. Clinical analysis of 247 children with whooping cough and the risk factors of severe cases. Chin J Pediatr. 2015;53(9):684–9. 26757969

[22] Akcay N, Tosun D, Bingol I, Bingol I, Citak A, Bayraktar S, Menentoglu ME, Sevketoglu E, Talip M, Umman Serin N, et al. Severe pertussis infections in pediatric intensive care units: a multicenter study. Eur J Pediatr. 2025;184(2):138. 39812867 10.1007/s00431-025-05978-0

[23] Kaczmarek MC, Ware RS, McEniery JA, Coulthard MG, Lambert SB. Epidemiology of pertussis-related paediatric intensive care unit (ICU) admissions in Australia, 1997–2013: an observational study. BMJ Open. 2016;6(4):e010386. 10.1136/bmjopen-2015-010386 PMC4823423 27053270

[24] Mbayei SA, Faulkner A, Miner C, Edge K, Cruz V, Pena SA, Kudish K, Coleman J, Pradhan E, Thomas S, et al. Severe Pertussis Infections in the United States, 2011–2015. Clin Infect Dis. 2019;69(2):218–26. 30321305 10.1093/cid/ciy889 PMC7108152

[25] Mack I, Erlanger TE, Lang P, Sinniger P, Perisa D, Heininger U. Dose-dependent effectiveness of acellular pertussis vaccine in infants: A population-based case-control study. Vaccine. 2020;38(6):1444–9. 31813648 10.1016/j.vaccine.2019.11.069

[26] Liu C, Yang L, Cheng Y, Xu H, Xu F. Risk factors associated with death in infants < 120 days old with severe pertussis: a case-control study. BMC Infect Dis. 2020;20(1):852. 33198647 10.1186/s12879-020-05535-0 PMC7668018

[27] Winter K, Zipprich J, Harriman K, Murray EL, Gornbein J, Hammer SJ, Yeganeh N, Adachi K, Cherry JD. Risk Factors Associated With Infant Deaths From Pertussis: A Case-Control Study. Clin Infect Dis. 2015;61(7):1099–106. 26082502 10.1093/cid/civ472

[28] Poeta M, Moracas C, Albano C, Petrarca L, Maglione M, Pierri L, Carta M, Montaldo P, Venturini E, De Luca M, et al. Pertussis outbreak in neonates and young infants across Italy, January to May 2024: implications for vaccination strategies. Euro Surveill. 2024;29(23). 10.2807/1560-7917.ES.2024.29.23.2400301 PMC11158011 38847118

[29] Scanlon K, Skerry C, Carbonetti N. Association of pertussis toxin with severe pertussis disease. Toxins (Basel). 2019;11(7). 10.3390/toxins11070373 PMC6669598 31252532

[30] Lin X, Zou J, Yao K, Li L, Zhong L. Analysis of antibiotic sensitivity and resistance genes of *Bordetella pertussis* in Chinese children. Med (Baltim). 2021;100(2):e24090. 10.1097/MD.0000000000024090 PMC10545409 33466172

[31] Fu P, Wang C, Tian H, Kang Z, Zeng M. *Bordetella pertussis* Infection in Infants and Young Children in Shanghai, China, 2016–2017: Clinical Features, Genotype Variations of Antigenic Genes and Macrolides Resistance. Pediatr Infect Dis J. 2019;38(4):370–6. 30882726 10.1097/INF.0000000000002160

[32] Hu Y, Zhou L, Du Q, Shi W, Meng Q, Yuan L, Hu H, Ma L, Li D, Yao K. Sharp rise in high-virulence *Bordetella pertussis* with macrolides resistance in Northern China. Emerg Microbes Infect. 2025;14(1):2475841. 40042368 10.1080/22221751.2025.2475841 PMC11921162

[33] Wang C, Li J, Chang H, Tian H, Cai J, Chen M, Wei Z, Zeng M. Levofloxacin is as effective as trimethoprim-sulfamethoxazole for the treatment of pertussis: a prospective observational study. J Microbiol Immunol Infect. 2026;59(1):117–23. 10.1016/j.jmii.2025.08.023 40957810

